# Editorial

**Published:** 1914-09

**Authors:** 


### EDITORIAL

The Business Office of the *Journal-Record* is 23 West Alabama Street.

The Editorial Office is 1014-15 Atlanta National Bank Building.

Address all Business Communications to *Journal-Record of Medicine,* 23 West Alabama Street.

Make remittances payable to *The Journal-Record of Medicine.*

On matters pertaining to the Editorial and Original Communications, address Edgar G. Ballenger, M. D., Atlanta, Ga.

Reprints of Original Articles will be furnished at cost price. Requests for the same should always be made in THE MANUSCRIPT.

We will present, postpaid, on request to each contributor of an original article, twenty (20) marked copies of *The Journal-Record of Medicine* containing such article.

### SCOPOLOMIN IN OBSTETRICS.

At Freiburg, Germany, some twelve years ago in the Frauenklinic, Kroenig and Gauss began the observation of the effect of this drug combined with morphin upon women in labor. Their experience has been known to the carful obstetricians of all lands almost since the beginning of their work and women have gone to Freiburg, repeatedly in some instances, from many different cities and countries for the relief from labor pains that they found there without danger to themselves or their babies.

Several progressive men who could afford the time have investigated the matter in this country during the interim, but never to the extent of recommending it to the Profession here nor of letting it be generally known that they had it under consideration.

Several months ago McClure's came out with a leading article describing the Daemmerschlaf or Twilight Sleep, in detail almost as if it were written for a medical magazine and handling it so completely that what once was looked upon as the delicacy that surrounded the lying-in chamber was completely removed and the public was invited to look upon and participate in the confinements in the neighborhood. In October still another article appears in that magazine blaming doctors for not taking up this method and bringing relief to all womankind in their travail.

The New York Medical Journal took a conservative stand as to this sort of publication and objected to introducing into public thought matters concerning which most of the readers could not be well enough informed to have definite opinions, while at the same time convincing the readers that all that was to be known was presented to them. They were given a little knowledge and a few facts and these the people were tempted to use rationally without the inhibition of the experience of the skilled and studious physician.

The New York Times took the matter up in its editorial page where it has previously considered affairs of public medicine fairly and in a broad-minded way. It took the Medical Journal to task, however, for what it said was the attempt to belittle or to put aside the advances made at Freiburg without proper investigations and also for the Journal's objection to such matters appearing in the public prints.

Thus there is a pretty discussion going on at present. Several physicians have either entered it or been brought into it. Ginsburg, of New York, wrote a letter to The Times saying that the method was not received with favor abroad outside of Freiburg and that he had been studying it in Berlin and Vienna. Rongy and Arluck, of New York, were quoted as to their opinions expressed at Medical meetings and in medical publications. Their experience seemed to be in favor of the process.

We are informed that there have been several cases in Atlanta, but at this writing no one cares to be quoted.

The Twilight Sleep means that at the beginning of labor small doses of scopolomin, combined with morphin, are given hypodermatically until the patient loses memory of what happens and also is free from pain. The test seems to be that when the patient is asked a question and the answer is given, she will not remember in a few minutes that the question was asked. This condition of amnesia must be reached and maintained by the drug and just such doses as are necessary to accomplish this are to be given if the patient can stand them. The result is a slow labor without pain. Nothing else is claimed for it. There may be as many tears of the parts as without it and it is said not to interfere with the use of instruments if they are demanded. The fundamental and essential fact in this treatment is that the physician shall be in actual constant attendance upon the patient until she is delivered. He alone can judge whether she has had enough or too much of the drug. There is no definite time of intermission between doses. The next thing or what is fully as important as the first, is that there should be absolute certitude as to the drug itself. Preparations vary and the alkaloid is unstable. It is chemically identical with Hyoscin. There is a question among many men if there is a difference therapeutically and if so, how the manufacturers can determine it. A secondary matter is the possible effect upon the child. About ten per cent of the Freiburg babies are cyanosed when born and have to receive special attention. The average time of labor in Rongy's 125 cases in this country was six and a half hours.

The question arises: Shall obstetricians take up this method and relieve 90% of the pains of labor?

The physician is worthy of his hire. There is no set of men so eager to relieve pain and suffering, so thoroughly imbued with the ambition to have success in life marked by the amount of practical good brought to others as that which sends its members into the homes and teaches them to come into the closest possible relations with the families. Still these men are worthy of their hire and they must live and be happy themselves in order to minister to others. It would shock the sense of any community to have even a strange physician say that he was indifferent to the pains of maternity. The people know better than they know anything else in this world that their doctor—the man who comes to their homes and helps them, no matter what the pain or distress, the man whose sympathy and advice is sought when all others seem as strangers—will do what is right if it is possible. But can they afford to have him? Can the doctor in the smaller towns where he knows the income of every man living near him, charge for his services in labor what this Daemmerschlaf demands? Let us say that he is seeing fifteen or twenty patients a day and travelling no little distance to get to them, which shall he slight in order to spend that long and constant time with one woman who is to have the Twilight Sleep? Or if he limits his practice to obstetrics and takes only such cases as he can well attend to and give each the time it needs, who is to pay him enough to make life successful for him?

Another factor of the treatment is the administration of pituitrin to hurry up the labor and overcome just this long delay. This, be it said, is an American modification. It goes to show that the doctors who are using it are trying to meet the conditions the questions outline.

It used to be taught by the splendidly skilled therapeutists of the last century that every drug administered, no matter how beneficial the sum total of its effect might be, was a poison and a foreign body in the physiologic metabolism we call health. And they were right. We look askance upon the filling of a woman in labor with three powerful and somewhat conflicting substances, and while we have the courage of the modern practitioner to do what seems best no matter what the danger and to take up new things that give reasonable promise of success, no matter what images are shattered thereby, we hesitate to approve of anything that will give even ten per cent of blue babies.

If the mothers that have been in travail and woeful pain to bring their daughters into the world were trained and permitted by our civilization to raise those daughters as they should, and if the fathers took care to keep themselves clean so that healthy daughters could be born to them, the succeeding generations would have slight need of the Daemmerschlaf.

We regret that the publicity of magazine and newspaper has been given until the subject could have been more thoroughly understood. Even the German Schlossingk who worked and studied two years at the Freiburg Klinic could not do his work well here because he did not understand American women. We could tell him without fear that he could spend a far longer time than that and at as great a number of foreign and native clinics as he might elect and yet not obtain that delectable degree of knowledge, that remarkable extent of comprehension. If that also is a necessary preliminary to the successful manipulation and administration of the Daemmerschlaf, it is indeed a sorry matter to create a state of unrest in the public mind over the possibility of banishing the pains of labor in this way.

